# TSC-22 inhibits CSF-1R function and induces apoptosis in cervical cancer

**DOI:** 10.18632/oncotarget.20296

**Published:** 2017-08-16

**Authors:** Min-Ji Cho, Ji-Yeon Lee, Min-Gwan Shin, Hyun-Ji Kim, Yu-Joung Choi, Seung Bae Rho, Boh-Ram Kim, Ik Soon Jang, Seung-Hoon Lee

**Affiliations:** ^1^ Department of Life Science, YongIn University, Yonginsi, Korea; ^2^ Research Institute, National Cancer Center, Goyang-si, Korea; ^3^ Division of Bioconvergence, Korea Basic Science Institute, Daejeon, Korea

**Keywords:** colony stimulating factor-1R, transforming growth factor-β-stimulated clone-22, cervical cancer, apoptosis

## Abstract

Colony stimulating factor 1 receptor (CSF-1R) regulates the monocyte/macrophage system, which is an essential component of cancer development. Therefore, CSF-1R might be an effective target for anti-cancer therapy. The overexpression of transforming growth factor (TGF)-β stimulated clone-22 (TSC-22) inhibits cancer cell proliferation and induces apoptosis, and TSC-22 is emerging as a key factor in tumorigenesis. In this study, we discovered CSF-1R as a new interacting partner of TSC-22 and identified its elevated expression in cervical cancer cells. In particular, we found that TSC-22 interacted with the intracellular tyrosine kinase insert domain (539–749) of CSF-1R, which activates the AKT and ERK signaling pathways. This binding blocked AKT and ERK signaling, thereby suppressing the transcriptional activity of NF-κB. The overexpression of TSC-22 significantly decreased CSF-1R protein levels, affecting their autocrine loop. TSC-22 injected into a xenograft mouse model of human cervical cancer markedly inhibited tumor growth. The reduction of CSF-1R protein significantly suppresses cervical cancer cell proliferation and motility and induces apoptotic cell death. This association between TSC-22 and CSF-1R could be used as a novel therapeutic target and prognostic marker for cervical cancer.

## INTRODUCTION

Transforming growth factor-β-stimulated clone-22 (TSC-22) was first isolated from mouse osteoblastic cells as a TGF-β –inducible gene. TSC-22 is a member of the leucine zipper transcription factor family and consists of 144 amino acids. TSC-22 is broadly expressed in almost all human tissues and organs, including the brain, liver, kidney, lung, prostate, testis, and ovary [1, 2]. Several studies reported the loss of TSC-22 expression in cancer and emphasized the functional role of the tumor suppressor gene. In salivary gland cancer, downregulation of TSC-22 led to cell differentiation [3]. In cervical cancer, TSC-22 expression inhibited cancer cell growth and promoted cellular apoptosis through the regulation of p53 ubiquitination [4].

To investigate other roles of TSC-22 in cervical cancer, we performed yeast two hybrid (Y2H) screening, and showed that CSF-1R directly interacts with TSC-22. Colony stimulating factor-1 receptor (CSF-1R), encoded by the c-*fms* proto-oncogene, is a class III transmembrane tyrosine kinase receptor. CSF-1 is a ligand and a primary growth factor involved in regulating the proliferation, survival, and differentiation of mononuclear phagocytes [5]. Previous studies revealed the importance of CSF-1/*CSF-1R* expression in various tumor types. Knockdown of CSF-1/*CSF-1R* increased apoptosis and reduced proliferation and migration of cervical cancer cells [6]. Overexpression of CSF-1 and CSF-1R stimulated invasion and metastasis in ovarian cancer [7].

CSF-1R is mainly composed of three parts: an extracellular ligand-binding domain, a transmembrane domain, and an intracellular tyrosine domain. CSF-1 activates CSF-1R tyrosine residues, leading to a subsequent phosphorylation cascade [8]. Inhibition of CSF-1R interrupted CSF-1 induced signaling, thus blocking ERK1/2 activity [9]. CSF-1/CSF-1R signaling promotes the proliferation of breast cancer cells through ERK1/2 phosphorylation [10]. Y721 phosphorylation in the intracellular domain of CSF-1R activated PI3K signaling and increased macrophage motility [11, 12]. Moreover, intracellular CSF-1R signaling not only regulates cancer cell survival and migration, but also stimulates CSF-1/CSF-1R, indicating that the expression of CSF-1 and CSF-1R is influenced by an autocrine loop [12, 13].

Here, we demonstrated the anti-proliferative effects of TSC-22 in cervical cancer cells and that TSC-22 induces apoptosis linked to interaction with CSF-1R. The novel molecular mechanism between TSC-22 and CSF-1R suggests a potential treatment for cervical cancer.

## RESULTS

### Interaction between TSC-22 and CSF-1R occurs in the cytoplasm

**Figure 1:**
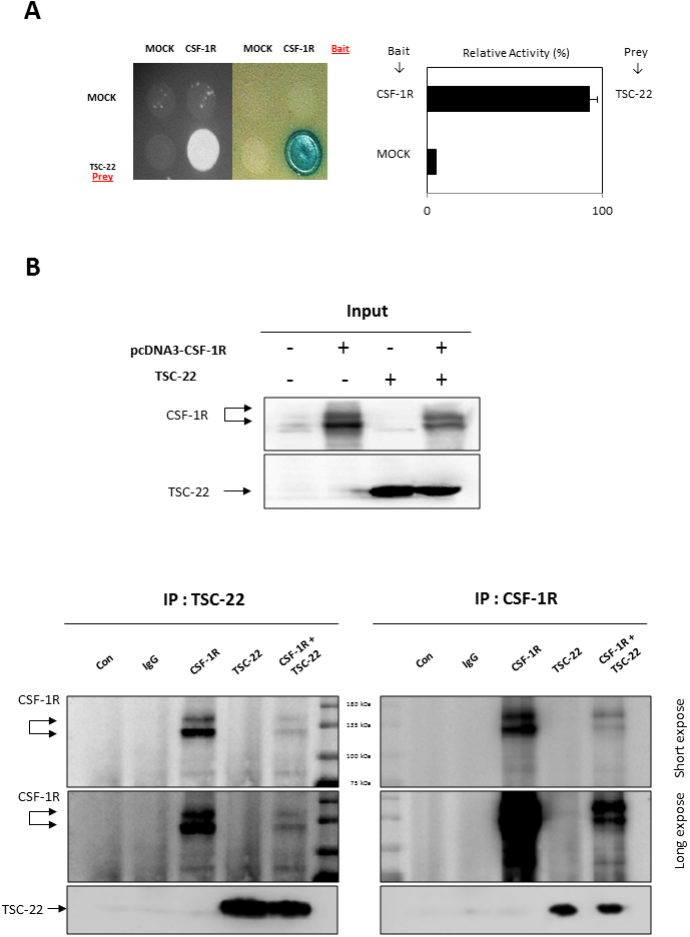
Interaction between TSC-22 and CSF-1R *in vivo* and *in vitro* **(A)** Transformants were assessed for their ability to grow on medium lacking leucine (left) and for β-galactosidase expression (right). Interaction between TSC-22 and CSF-1R was determined by β-galactosidase expression. **(B)** HEK293a cells were transfected with pcDNA4-CSF-1R or pcDNA4-TSC-22. After 2 days, cell lysates were immunoprecipitated with CSF-1R antibody or TSC-22 antibody. IP samples were analyzed by western blot.

**Figure 2:**
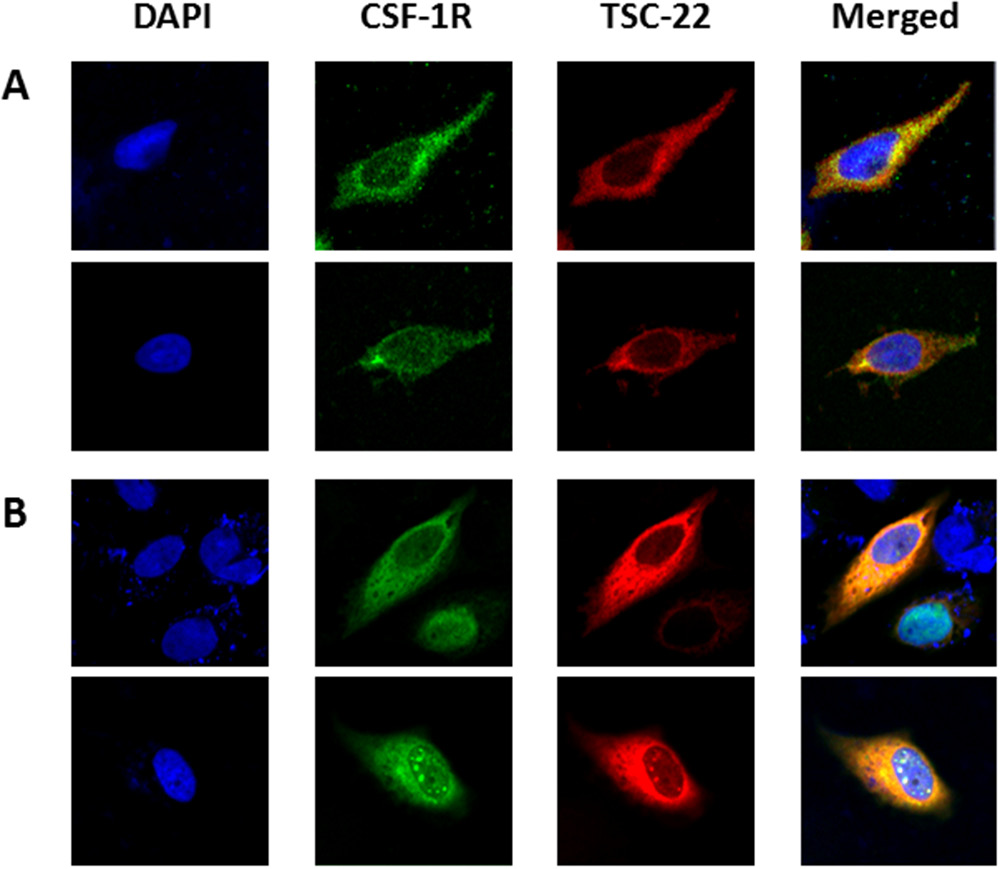
Localization of CSF-1R and TSC-22 HeLa cells were non-transfected **(A)** or co-transfected with pcDNA4-TSC-22 and CSF-1R **(B)**. ICC was performed using CSF-1R (green) and TSC-22 (red) antibodies, and their co-localization was represented by a yellow color. Cell images were taken using a confocal fluorescence microscope (magnification ×400).

To discover new functions of TSC-22, we performed Y2H screening. CSF-1R was selected as a TSC-22 binding protein (data not shown). The interaction of TSC-22 with CSF-1R was verified by growth assay and β-galactosidase assay *in vivo* (Figure 1A). To confirm binding between TSC-22 and CSF-1R *in vitro*, HEK293a cells were transfected with TSC-22 and CSF-1R plasmids. Cell lysates were immunoprecipitated with CSF-1R or TSC-22 antibodies and immunoblotted (Figure 1B). Figure 2A and 2B shows that TSC-22 binds strongly to CSF-1R. To clarify the localization of CSF-1R and TSC-22, we performed immunocytochemistry. Figure 2 shows that both TSC-22 and CSF-1R were present in the cytoplasm. Therefore, the interaction between TSC-22 and CSF-1R occurs in the cytoplasm.

### TSC-22 induces apoptosis in cervical cancer cells

**Figure 3:**
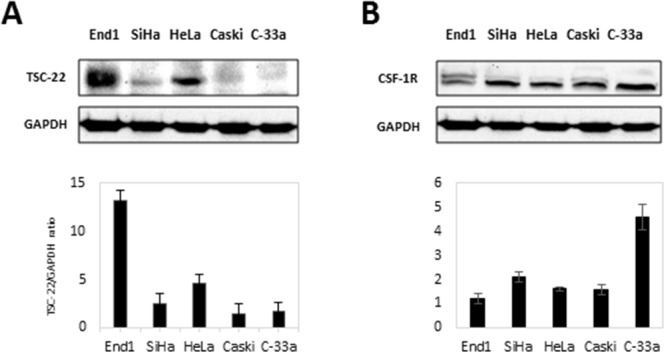
Protein levels of CSF-1R and TSC-22 in cervical cancer cells **(A)** Total protein was prepared from each indicated cell line. The expression of TSC-22 protein was analyzed using western blotting. Expression levels of TSC-22 were normalized to GAPDH expression. **(B)** Whole-cell lysates were prepared from the same cell line indicated in A and used in western blot analysis. CSF-1R expression levels are presented as the CSF-1R/GAPDH ratio and compared with a normal cervical cell line (End1).

Previous studies reported that TSC-22 is downregulated in cervical cancer tissues [4], so we compared the expression levels of TSC-22 in cervical cancer cell lines (SiHa, HeLa, Caski, C-33a) with a normal cervical cell line (End1). Data revealed that TSC-22 was greatly decreased in cervical cancer cells compared to normal cells (Figure 3A). In contrast, we determined that CSF-1R expression was upregulated in cervical cancer cell lines compared to the normal cervical cell line (Figure 3B). These results suggest that downregulation of TSC-22 and upregulation of CSF-1R may be involved in carcinogenesis in cervical cancer.

**Figure 4:**
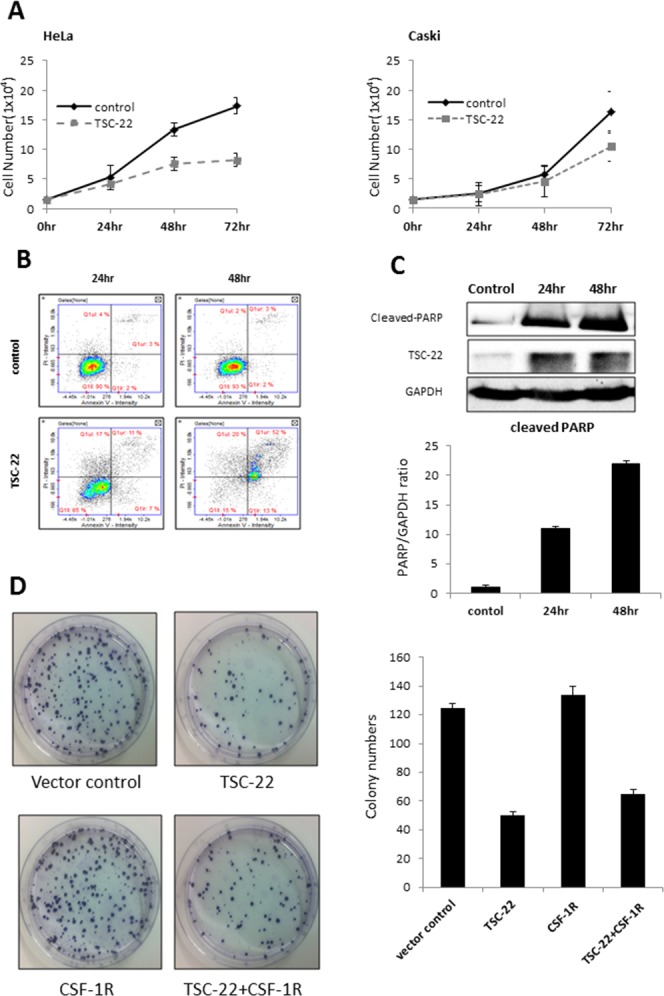
Pro-apoptotic role of TSC-22 in cervical cancer cells **(A)** HeLa and Caski cells were plated in 24-well plates and transiently transfected with pcDNA4 or pcDNA4-TSC-22. The number of surviving cells was counted under a microscope. **(B)** HeLa cells were transiently transfected with pcDNA4 or pcDNA4-TSC-22. After the indicated time points, AnnexinV/PI double staining was performed to quantify apoptosis of HeLa cells using flow cytometry. **(C)** Protein level of cleaved PARP was analyzed using western blotting of control and TSC-22 overexpressing cells. **(D)** TSC-22- or CSF-1R-transfected HeLa cells were re-plated in 100-mm dishes and cultured for 14 days. The number of crystal violet stained colonies was counted.

We examined whether overexpression of TSC-22 inhibits tumor cell growth. We counted the number of viable HeLa and Caski cells transfected with control vector or TSC-22 expression vector every 24 hours. As shown in Figure 4A, the growth curve of TSC-22 transfected cells was dramatically decreased. We also performed flow cytometry of HeLa cells to verify apoptotic cell death. The apoptotic rate (%) increased significantly in TSC-22 transfected cells compared with empty vector transfected cells (Figure 4B). To confirm the previous apoptosis results, cleaved PARP protein levels, a marker of apoptosis, were examined using western blotting. The overexpression of TSC-22 increased cleaved PARP levels in a time-dependent manner (Figure 4C). These results indicate that TSC-22 retards cell growth and induces apoptosis in cervical cancer cells.

We also examined colony formation ability. Figure 4D shows that both the number and size of colonies formed after 14 days were significantly reduced in TSC-22-transfected HeLa cells, but elevated in CSF-1R-transfected cells compared to vector control. Similarly, TSC-22 and CSF-1R co-transfected cells formed twofold fewer colonies than CSF-1R only transfected cells. Hence, TSC-22 inhibits cancer cell proliferation induced by CSF-1R.

### Binding regions between TSC-22 and CSF-1R

**Figure 5:**
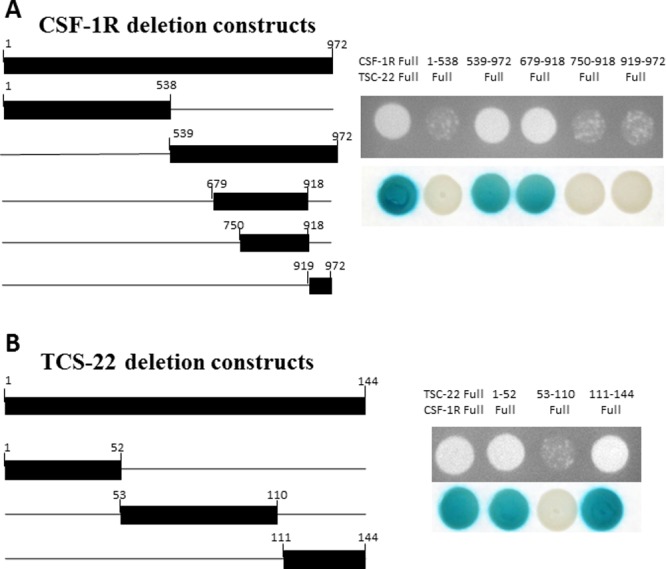
Mapping of the binding regions between TSC-22 and CSF-1R Left schematic diagram displays the cDNA deletion constructs of CSF-1R and TSC-22. Right panel shows the results of protein-protein interaction using growth and β-galactosidase assays. Positive transformants are indicated by the formation of colonies on the plate.

We next investigated how TSC-22 regulates CSF-1R signaling. We made deletion constructs for TSC-22 and CSF-1R to define the specific binding region between TSC-22 and CSF-1R. Their interaction was analyzed using Y2H assays. TSC-22 bound to the region between 539 and 749 amino acids in CSF-1R (Figure 5A). On the other hand, CSF-1R bound to the regions from 1 to 52 and 111 to 144 amino acids in TSC-22 (Figure 5B). These results indicate that TSC-22 interacts directly with the intracellular tyrosine kinase insert domain (539–749) of CSF-1R.

### TSC-22 blocks the CSF-1R-dependent signaling pathway

**Figure 6:**
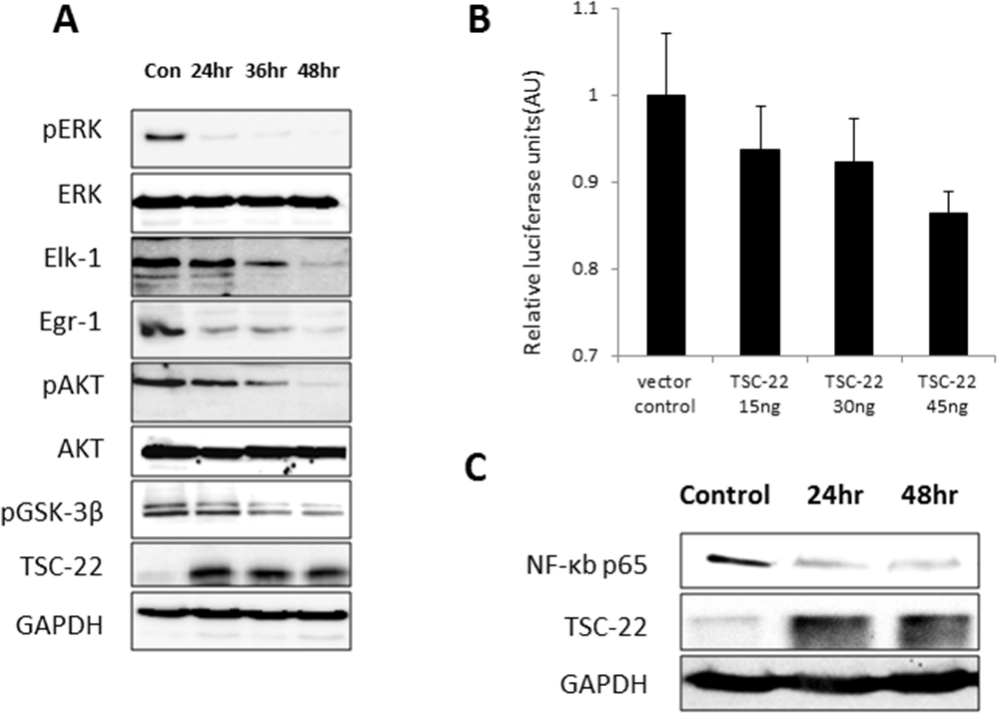
Inhibition of AKT and ERK signaling pathways and transcriptional activity of NF-κB by TSC-22 **(A)** HeLa cells were transiently transfected with pcDNA4-TSC-22, then harvested at the indicated time points and analyzed by western blot. The expression levels of pERK, ERK, Elk-1, Egr-1, pAKT, AKT, pGSK-3β, and GAPDH were measured. **(B)** HeLa cells were transiently transfected with TSC-22 in a dose-dependent manner. The activity of the NF-κB promoter was measured using a luciferase reporter gene assay. The firefly luciferase and Renilla luciferase values were determined. Firefly luciferase levels were normalized to Renilla luciferase levels. **(C)** TSC-22-overexpressing cells were harvested at 24-hour intervals and NF-κB protein levels were evaluated using western blotting.

We hypothesized that TSC-22 affects the CSF-1R signaling pathway because of its interaction with the intracellular tyrosine kinase insert domain (539–749). We analyzed levels of several proteins that mediate CSF-1/CSF-1R signaling in TSC-22-overexpressing cells. We demonstrated that TSC-22 reduces the phosphorylation of ERK and AKT, leading to a decline in their downstream proteins, including GSK-3β, Elk-1, and Egr-1 (Figure 6A). CSF-1R is a receptor activator of the NF-κB promoter through the ERK and AKT pathways [14]. We next performed luciferase assay and western blotting for NF-κB. We found that TSC-22 suppressed the transcriptional activity of NF-κB and reduced protein levels in dose- and time-dependent manners (Figure 6B, 6C). Therefore, TSC-22 inhibits the CSF-1/CSF-1R signaling cascade.

**Figure 7:**
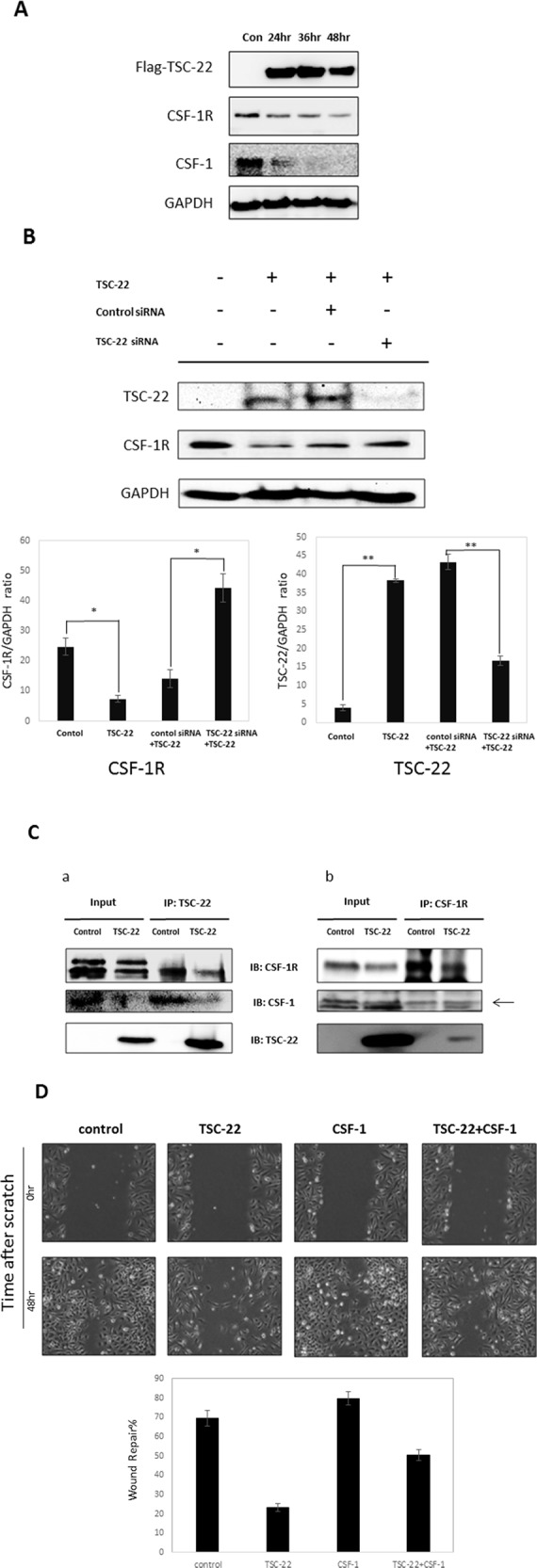
The effect of TSC-22 in the CSF-1R autocrine loop **(A)** HeLa cells were transfected with TSC-22 in a time-dependent manner. Whole cell lysates were used in western blotting. The expression levels of TSC-22, CSF-1R, CSF-1, and GAPDH are shown. **(B)** HeLa cells were transfected with control siRNA or TSC-22 siRNA for 24 hours and then transfected with the TSC-22 expression vector for 24 hours. Protein levels of CSF-1R and TSC-22 were determined by western blot assay. **(C)** Cell lysates were immunoprecipitated with TSC-22 or CSF-1R antibody and immunoblotted with CSF-1R, TSC-22, or CSF-1. **(D)** HeLa cells were transiently transfected with pcDNA4-TSC-22 and pcDNA4-CSF-1. An injury line was made on the confluent monolayer of cells. Cellular migration was observed with a light microscope (×40) at the indicated time points. The width of the injury line was measured and plotted on a graph.

Furthermore, TSC-22 decreased the expression of CSF-1R and CSF-1 (Figure 7A). When cells overexpressing TSC-22 were transfected with TSC-22 siRNA, the CSF-1R expression level increased (Figure 7B). We predicted that TSC-22 interrupts the interaction between CSF-1R and CSF-1. We preformed co-immunoprecipitation to confirm their binding. As shown in Figure 7C, TSC-22 decreased the protein levels of CSF-1R and CSF-1 in immunoprecipitated samples. However, TSC-22 did not block their interaction, because the expression levels of CSF-1R and CSF-1 were also reduced by TSC-22 in whole lysates. These results revealed that TSC-22 inhibits the ERK/AKT/NF-κB signaling pathway, decreasing the expression of CSF-1R and CSF-1.

We performed a wound healing assay in HeLa cells. TSC-22 significantly reduced cell migration (Figure 7D). These results indicate that TSC-22 interrupts CSF-1 signals mediated by CSF-1R and suppresses cervical cancer cell proliferation and migration.

### TSC-22 inhibits cervical cancer cell growth *in vivo*

**Figure 8:**
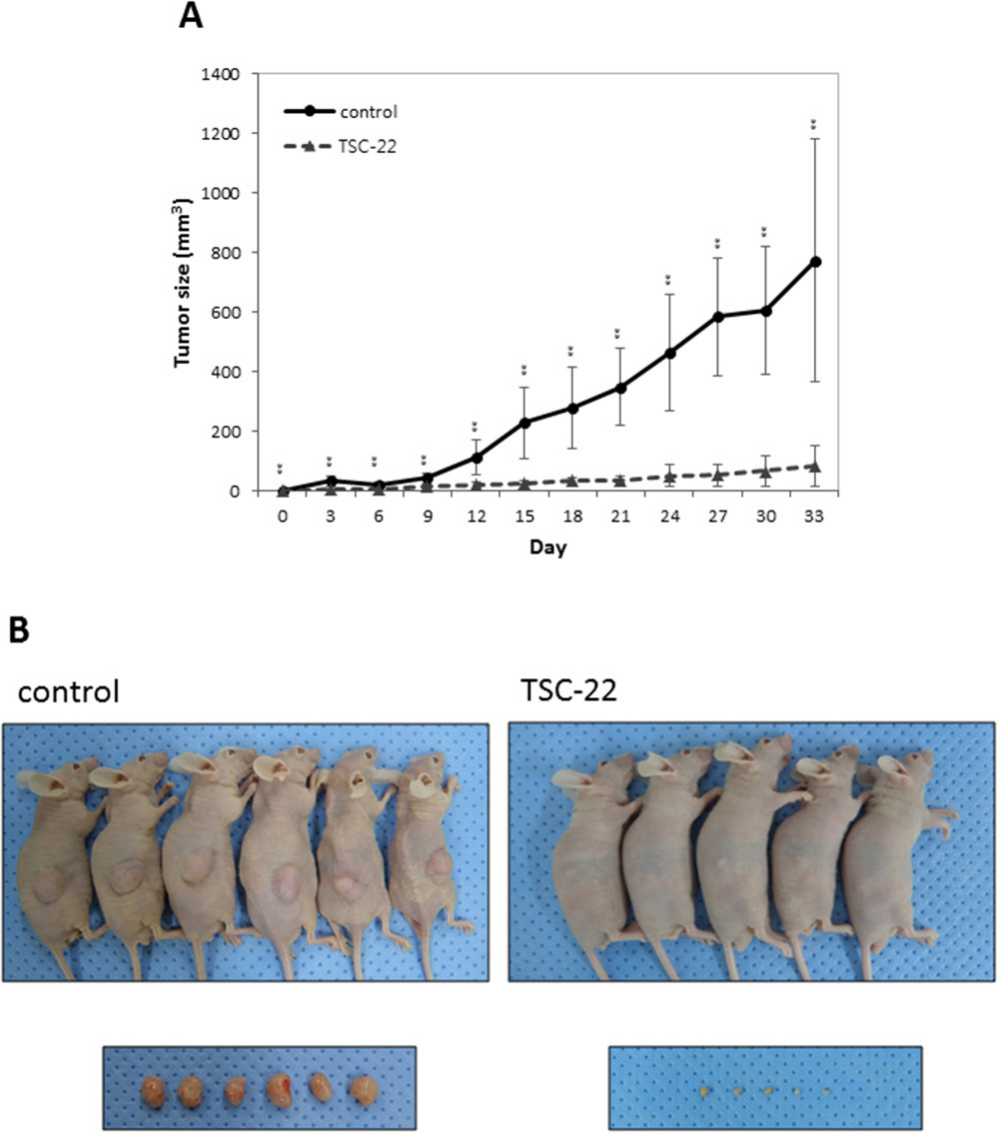
TSC-22 inhibits cervical cancer cell growth *in vivo* Control or TSC-22 transfected HeLa cells were injected subcutaneously into nude mice. Tumor size was measured every 3 days and plotted on a line graph. Images were taken at the time of dissection.

To assess whether TSC-22 inhibits cervical cancer cell growth *in vivo*, HeLa cells were transfected with TSC-22 expression vector or empty vector. The transfected cells were injected subcutaneously into immune-deficient BALB/c nude mice. The tumor volume was monitored for 33 days. As shown in Figure 8, TSC-22 transfected cells formed smaller tumor masses than the control. These results indicate that TSC-22 can be used as a tumor suppressor gene in cervical cancer therapy.

## DISCUSSION

Cancer is generally caused by the alteration of regulatory genes. Oncogenes or tumor suppressor genes are expressed abnormally compared to normal genes. Therefore, it is critical in cancer treatment to learn how they control cancer cell proliferation, differentiation, and survival. Previous studies have reported low levels of TSC-22 in various cancers, such as cervical [4], salivary gland [15–17], prostate [18], and brain tumor [19] cancers. TSC-22 plays a role as a tumor suppressor gene, but the detailed mechanism of TSC-22 remains elusive. Therefore, we screened TSC-22 binding proteins to discover new functions of TSC-22 and identified CSF-1R as a novel interacting partner. Many studies have demonstrated a relationship between the abnormal expression of CSF-1/CSF-1R and tumor development. Overexpression of CSF-1R was observed in many human cancer types, including prostate [20], breast [21], and hepatocellular carcinoma [22, 23]. The upregulation of CSF-1R expression was associated with macrophage growth in breast and prostate carcinomas [24]. In addition, overexpression of CSF-1R was related to chemotherapy resistance in lung cancer. High expression of CSF-1R indicates a poor cancer prognosis [25]. We demonstrated elevated CSF-1R expression contrary to decreased expression of TSC-22 in cervical cancer cells, consistent with the above-mentioned studies. Moreover, TSC-22 significantly retarded cervical cancer cell growth and induced apoptosis in HeLa and Caski cells. In addition, TSC-22 reduced the number of colonies induced by CSF-1R. Therefore, TSC-22 inhibits the effect of CSF-1 on cell proliferation and death by direct interaction.

To further explore how TSC-22 affects CSF-1R, we investigated the specific binding region between TSC-22 and CSF-1R. CSF-1R consists of 972 amino acids with three main regions. The extracellular segment is a ligand-binding domain (residues 1–512). The membrane spanning region is a transmembrane domain (residues 513–537). The intracellular domain contains a tyrosine kinase domain (residues 538–972), which phosphorylates tyrosine residues [8]. Our data revealed that TSC-22 interacts with amino acids 539–749, the intracellular tyrosine kinase domain. The intracellular domain of CSF-1R is essential for macrophage proliferation and differentiation. Recent studies suggest that the CSF-1/CSF-1R signaling system regulates proliferation, differentiation, and survival of cells via the ERK pathway and Akt pathway. Overexpression of CSF-1R activates the ERK1/2 signaling pathway leading to the stimulation of proliferation [26]. Phosphorylation of CSF-1R Y721 enhances tumor cell motility and invasion through the PI3K/AKT pathway [11, 12, 27]. CSF-1R inhibitors block CSF-1-regulated signaling (ERK1/2, AKT) [28]. Hence, we hypothesized that TSC-22 interrupts the CSF-1R signaling pathway by direct interaction, instead of through several tyrosine kinases. We analyzed the protein levels in the ERK and AKT signaling pathways and determined that overexpression of TSC-22 decreased phosphorylation of ERK and AKT and deregulated the downstream signaling. Moreover, phosphorylation of p65 NF-κB depends on the ERK signaling pathway. AKT induces NF-κB transcriptional activity by inhibiting the interaction between NF-κB and IκB and subsequent degradation of IκB [29–31]. Using a luciferase assay, we demonstrated that TSC-22 decreases transcription of NF-κB via regulation of ERK and Akt (Figure 6A). A western blot assay also demonstrated that NF-κB protein expression is reduced by TSC-22 (Figure 6C). Therefore, TSC-22 reduced both the transcriptional and translational levels of NF-κB.

Our data revealed that TSC-22 decreased CSF-1R and CSF-1 expression, likely through NF-κB as a transcriptional factor of CSF-1R [32]. However, these effects led to another question: whether TSC-22 influences the interaction between CSF-1R and CSF-1, as TSC-22 may interact with CSF-1R instead of CSF-1. Interestingly, CSF-1R and CSF-1 proteins in immunoprecipitated samples were decreased and the expression levels of both CSF-1R and CSF-1 in whole cell lysates were reduced. Therefore, TSC-22 did not affect the binding between CSF-1R and CSF-1. CSF-1R not only mediates the ERK/AKT pathway, but is also regulated by the ERK/AKT dependent pathway [33, 34]. CSF-1R also binds the promoter of CSF-1 thereby inducing CSF-1 expression. CSF-1R and CSF-1 are modulated by the autocrine system contributing to aggressive cancers [13, 35, 36]. We inferred that TSC-22 inhibits CSF-1R and CSF-1 expression by blocking the CSF-1R-dependent signaling pathway. However, understanding the precise mechanisms of interaction between TSC-22 and CSF-1 requires further investigation.

In conclusion, we propose TSC-22 as a negative regulator of CSF-1R or the CSF-1/CSF-1R signaling pathway. TSC-22 inhibits CSF-1R-dependent signaling by direct interaction, and decreases cervical cancer proliferation and migration. Our findings imply that TSC-22 targeting CSF-1R signaling may have therapeutic effect in cervical cancer.

## MATERIALS AND METHODS

### Cell culture and transfection

All cell lines were incubated at 37°C in a humidity 5% CO2 incubator, fed every 2-3 days with complete medium and passed when cells are in the confluent state. HEK293a, HeLa, Caski, SiHa, C-33A cell line were cultured in recommended medium supplemented with 10% fetal bovine serum (FBS, WelGENE) and 1% Amphotericin B/Streptomycin/Penicillin (Gibco). Cell transfection was performed using the X-tremeGENE HP reagent (Roche Applied Science) according to the manufacturer's instructions.

### Construction of plasmids

pSM-CSF-1R plasmid was provided by Dr.Martine Roussel, St. Jude Children's Research Hospital and inserted in pcDNA4 vector using *EcoRI* restriction site. pOTB7-CSF-1 plasmid was provided from from Korea Human Gene Bank, Medical Genomics Research center, KRIBB, Korea. CSF-1R expression vectors were constructed by cloning full-length CSF-1R into pcDNA4 and pcDNA3- flag vector using *EcoRI* and *BamHI* restriction sites. CSF-1 expression vector were provided by Dr. Ghanshyam Swarup, Council of Scientific and Industrial Research, Hyderabad, India.

### Yeast two hybrid analysis

The EGY48 yeast strain was used in present study and Matchmaker LexA Two-Hybrid system (Clontech, Palo Alto, CA) was used to perform the yeast two-hybrid assay according to the manufacture's instructions. The wild type TSC-22 were amplified by PCR and cloned into pGilda vector using *EcoRI* and *Xho I* restriction sites and used as baits. Human cDNA library was inserted in pB42AD prey vector for the yeast-two hybrid screening. The wild type and deleted CSF-1R were amplified by PCR and cloned into pGilda vector using *EcoRI* and *BamHI* restriction sites and used as baits. Also, the wild type and deleted TSC-22 were amplified by PCR and cloned into pB42AD prey vector between *EcoR I* and *Xho I* restriction sites for the growth and ß-galactosidase assay. The primers used for amplication are shown in the Table 1. Bait and prey vectors were co-transformed in EGY48 yeast strain and transformants were grown for 3 days at 30°C on plates in dropout media lacking uracil, histidine and tryptophan. Positive colonies were confirmed by growth and β-galactosidase assay on plates lacking uracil, histidine, tryptophan and leucine or containing X-gal, respectively.

**Table 1:** Primers used in cloning of yeast two-hybrid assay

Gene	Location	Forward/reverse	Primer sequence	Restriction site
TSC-22	Full 1-144	Forward	5’-CGGGAATTCATGAAATCCCAATGGTGT-3‘	*EcoR I*
		Reverse	5’-CGGCTCGAGCTATGCGGTTGGTCCTGA-3’	*Xho I*
	1-52	Forward	5’-CGGGAATTCATGAAATCCCAATGGTGT-3’	*EcoR I*
		Reverse	5’-ATTCTCGAGTCAGTCAATAGCTACCAC-3’	*Xho I*
	53-110	Forward	5’-CGGGAATTCATGAACAAAATCGAGCAAGCT-3’	*EcoR I*
		Reverse	5’-ATTCTCGAGTCAAAACTGGGCAAGCTG-3’	*Xho I*
	111-144	Forward	5’-CGGGAATTCATGCAGGCCCAGCTGCAGACT-3’	*EcoR I*
		Reverse	5’-CGGCTCGAGCTATGCGGTTGGTCCTGA-3’	*Xho I*
CSF-1R	Full 1-972	Forward	5’-CGGGAATTCATGGGCCCAGGAGTTC-3’	*EcoR I*
		Reverse	5’-ATGCGGATCCTCAGCAGAACTGATA-3’	*BamH I*
	1-538	Forward	5’-CGGGAATTCATGGGCCCAGGAGTTC-3’	*EcoR I*
		Reverse	5’-ATGCGGATCCTCAGTACAATAGCAG-3’	*BamH I*
	539-972	Forward	5’-ATGCGAATTCATGAAGTATAAGCAG-3’	*EcoR I*
		Reverse	5’-ATGCGGATCCTCAGCAGAACTGATA-3’	*BamH I*
	679-918	Forward	5’-ATGCGAATTCATGGAGGCCATGCTG-3’	*EcoR I*
		Reverse	5’-ATGCGGATCCTCACTCCTGTCCTCT-3’	*BamH I*
	750-918	Forward	5’-ATGCGAATTCATGGAGCTCCGGGAC-3’	*EcoR I*
		Reverse	5’-ATGCGGATCCTCACTCCTGTCCTCT-3’	*BamH I*
	919-972	Forward	5’-ATGCGAATTCATGGAGCGGGACTAT-3’	*EcoR I*
		Reverse	5’-ATGCGGATCCTCAGCAGAACTGATA-3’	*BamH I*

### AnnexinV/PI staining

HeLa cells were plated into 6-well plates and transfected with expression vectors. Transfected cells were havested with Detachin solution at different time point and pelleted by centrifugation. Each of the cells was washed once with 1X PBS and resuspended in 100 μl of Annexin V binding buffer. Annexin V-FITC and Hoechst 33342 were then added to cell suspension. After the incubation at 37°C for 15min, cells were washed with Annexin V binding buffer, stained with 2 μl of PI (propodium iodide, 500 μg/ml) and immediately analyzed with NucleoCounter® NC-3000^TM^.

### Western blotting

Cells were lysed in RIPA buffer (50mM Tris-Cl pH 7.5, 150mM NaCl, 1% NP-40, 0.5% sodium deoxycholate, 0.1% SDS, 5mM PMSF) for 30min on ice. The extracts were centrifuged at 13,000rpm for 10min at 4°C, and the protein concentration was determined using the Bradford assay.

Protein extracts were separated by SDS-PAGE using 8 to 12% polyacrylamide gel and transferred onto a nitrocellulose membrane (Bio-Rad, 0.45μM). The membranes were blocked for 1hr in 5% skim milk and incubated at 4°C for overnight with specific primary antibody. Antibodies against TSC-22 (sc-101195), CSF-1(sc-1324), CSF-1R(sc-692), pERK (sc-7383), GSK-3β (sc-8257) and GAPDH (sc-25778) were purchased from Santa Cruz Biotechnology. Antibodies against pAKT (#4060) and cleaved PARP (#9541) were purchased from Cell signaling. Antibodies against Akt (1085-1) and flag (F1804) were purchased from EPIT-MICS and Sigma aldrich, respectively. Following primary antibody incubation, the membranes were washed three times for 5min in 1X TBS-T, incubated for 1hr with horseradish peroxidase-conjugated secondary antibody. Immunoreactivity was detected using ECL chemiluminescent solution (advansta) and exposed by Chemidoc (Bio-Rad).

### Immunocytochemistry

Cells were grown on 18mm diameter cover glass (Marienfeld). After 48hr incubation, the cells were rinsed twice with 1X PBS and fixed and permeabilized in methanol-acetone mixture (1:1) for 7min at −20°C. Fixed cells were blocked with 5% BSA/PBS-T (PBS, 0.2% Tween-20) for 1hr at room temperature, and then incubated with CSF-1R antibody (1:250 dilution) and TSC-22 antibody (1:500 dilution) for 16hrs at 4°C. The cells were washed three times with 1X PBS for 5min each and incubated with alexa fluor 488 goat anti-rabbit IgG (Green) and alexa fluor 568 goat anti-mouse IgG (Red) antibody (Invitrogen, 250:1 dilution) in darkness for 1hr at room temperature. Finally, the cells were counterstained with 1μg/ml DAPI for 1min at room temperature and mounted on slides. The signals and co-localization were detected using the confocal fluorescence microscopy

### Coimmunoprecipitations

Cells were lysed in NP-40 lysis buffer (20mM Tris-HCl pH8.0, 150mM NaCl, 1% Nonident P-40, 1mM PMSF) for 30min on ice. Extracts were centrifuged at 13,000rpm for 10min at 4°C, and the protein concentration was measured using the Bradford assay. Each cell lysate (1.5mg) was incubated with CSF-R antibody (Santa cruz) or TSC-22 antibody (Santa curz) for overnight at 4°C. Following incubation, protein was immunoprecipitated using protein A/G agarose beads (Santa cruz) for 3hr at 4°C with gently rotation. The immunoprecipitates was washed three times with lysis buffer and boiled in 40 μl of 1X SDS sample buffer for 5min at 95°C. After centrifugation, the supernatant was analyzed by Western blot.

### Colony formation assay

1×10^5^ HeLa cells were seeded on 6-well plates and transfected. At 8hr after transfection, one thousand cells (1×10^3^) were re-plated onto 100mm plate in triplicate and cultured for 14 days to allow colonies to form. Then the media were removed and colonies were stained with 0.5% crystal violet solution (in distilled water) at room temperature for 15min. The plates were rinsed with water and colonies were counted. Values are expressed as mean ±SD from two experiments.

### Luciferase assays

1.3×10^4^ HeLa cells were seeded on 24-well plates and transfected. At 24hr after transfection, The cells were harvested and lysed in lysis buffer provided by manufacturer (promega). Cell extracts were used for the luciferase assay system (promega). Values are the means and standard deviations of results from three independent experiments.

### S.C. tumor models

To establish tumors in mice, TSC-22 expressing pcDNA3 vector-transfected 1 × 10^6^ HeLa cells were injected s.c. in the middorsal region of BALB/c nude mice 5-6 per group). Tumor size was evaluated using caliper measurements every 3 days. Mice were killed on day 33, and tumors were excised.

### Data analysis and statistics (statistical analysis)

Values are presented as the mean ± SD or ± SE. Statistical comparisons between groups were performed using the Student's *t*-test. *p* < 0.05 was considered statistically significant.

## References

[1] Kester HA, Ward-van Oostwaard TM, Goumans MJ, van Rooijen MA, van Der Saag PT, van Der Burg B, Mummery CL (2000). Expression of TGF-beta stimulated clone-22 (TSC-22) in mouse development and TGF-beta signalling. Dev Dyn.

[2] Khoury CM, Yang Z, Li XY, Vignali M, Fields S, Greenwood MT (2008). A TSC22-like motif defines a novel antiapoptotic protein family. FEMS Yeast Res.

[3] Kawamata H, Fujimori T, Imai Y (2004). TSC-22 (TGF-beta stimulated clone-22): a novel molecular target for differentiation-inducing therapy in salivary gland cancer. Curr Cancer Drug Targets.

[4] Yoon CH, Rho SB, Kim ST, Kho S, Park J, Jang IS, Woo S, Kim SS, Lee JH, Lee SH (2012). Crucial role of TSC-22 in preventing the proteasomal degradation of p53 in cervical cancer. PLoS One.

[5] Sherr CJ (1990). Colony-stimulating factor-1 receptor. Blood.

[6] Kirma N, Hammes LS, Liu YG, Nair HB, Valente PT, Kumar S, Flowers LC, Tekmal RR (2007). Elevated expression of the oncogene c-fms and its ligand, the macrophage colony-stimulating factor-1, in cervical cancer and the role of transforming growth factor-beta1 in inducing c-fms expression. Cancer Res.

[7] Chambers SK (2009). Role of CSF-1 in progression of epithelial ovarian cancer. Future Oncol.

[8] Pixley FJ, Stanley ER (2004). CSF-1 regulation of the wandering macrophage: complexity in action. Trends Cell Biol.

[9] Huynh J, Kwa MQ, Cook AD, Hamilton JA, Scholz GM (2012). CSF-1 receptor signalling from endosomes mediates the sustained activation of Erk1/2 and Akt in macrophages. Cell Signal.

[10] Morandi A, Barbetti V, Riverso M, Dello Sbarba P, Rovida E (2011). The colony-stimulating factor-1 (CSF-1) receptor sustains ERK1/2 activation and proliferation in breast cancer cell lines. PLoS One.

[11] Sampaio NG, Yu W, Cox D, Wyckoff J, Condeelis J, Stanley ER, Pixley FJ (2011). Phosphorylation of CSF-1R Y721 mediates its association with PI3K to regulate macrophage motility and enhancement of tumor cell invasion. J Cell Sci.

[12] Cioce M, Canino C, Goparaju C, Yang H, Carbone M, Pass HI (2014). Autocrine CSF-1R signaling drives mesothelioma chemoresistance via AKT activation. Cell Death Dis.

[13] Patsialou A, Wyckoff J, Wang Y, Goswami S, Stanley ER, Condeelis JS (2009). Invasion of human breast cancer cells in vivo requires both paracrine and autocrine loops involving the colony-stimulating factor-1 receptor. Cancer Res.

[14] Kalbasi Anaraki P, Patecki M, Tkachuk S, Kiyan Y, Haller H, Dumler I (2015). Urokinase receptor mediates osteoclastogenesis via M-CSF release from osteoblasts and the c-Fms/PI3K/Akt/NF-κB pathway in osteoclasts. J Bone Miner Res.

[15] Nakashiro K, Kawamata H, Hino S, Uchida D, Miwa Y, Hamano H, Omotehara F, Yoshida H, Sato M (1998). Down-regulation of TSC-22 (transforming growth factor beta-stimulated clone 22) markedly enhances the growth of a human salivary gland cancer cell line in vitro and in vivo. Cancer Res.

[16] Uchida D, Kawamata H, Omotehara F, Miwa Y, Hino S, Begum NM, Yoshida H, Sato M (2000). Over-expression of TSC-22 (TGF-beta stimulated clone-22) markedly enhances 5-fluorouracil-induced apoptosis in a human salivary gland cancer cell line. Lab Invest.

[17] Kawamata H, Nakashiro K, Uchida D, Hino S, Omotehara F, Yoshida H, Sato M (1998). Induction of TSC-22 by treatment with a new anti-cancer drug, vesnarinone, in a human salivary gland cancer cell. Br J Cancer.

[18] Rentsch CA, Cecchini MG, Schwaninger R, Germann M, Markwalder R, Heller M, van der Pluijm G, Thalmann GN, Wetterwald A (2006). Differential expression of TGFbeta-stimulated clone 22 in normal prostate and prostate cancer. Int J Cancer.

[19] Shostak KO, Dmitrenko VV, Garifulin OM, Rozumenko VD, Khomenko OV, Zozulya YA, Zehetner G, Kavsan VM (2003). Downregulation of putative tumor suppressor gene TSC-22 in human brain tumors. J Surg Oncol.

[20] Ide H, Seligson DB, Memarzadeh S, Xin L, Horvath S, Dubey P, Flick MB, Kacinski BM, Palotie A, Witte ON (2002). Expression of colony-stimulating factor 1 receptor during prostate development and prostate cancer progression. Proc Natl Acad Sci U S A.

[21] Kluger HM, Dolled-Filhart M, Rodov S, Kacinski BM, Camp RL, Rimm DL (2004). Macrophage colony-stimulating factor-1 receptor expression is associated with poor outcome in breast cancer by large cohort tissue microarray analysis. Clin Cancer Res.

[22] Yang DH, Huang W, Cui J, Shu JC, Tang SH, Zhang WJ, Liang JH (2004). The relationship between point mutation and abnormal expression of c-fms oncogene in hepatocellular carcinoma. Hepatobiliary Pancreat Dis Int.

[23] Jia JB, Wang WQ, Sun HC, Zhu XD, Liu L, Zhuang PY, Zhang JB, Zhang W, Xu HX, Kong LQ, Lu L, Wu WZ, Wang L, Tang ZY (2010). High expression of macrophage colony-stimulating factor-1 receptor in peritumoral liver tissue is associated with poor outcome in hepatocellular carcinoma after curative resection. Oncologist.

[24] Strachan DC, Ruffell B, Oei Y, Bissell MJ, Coussens LM, Pryer N, Daniel D (2013). CSF1R inhibition delays cervical and mammary tumor growth in murine models by attenuating the turnover of tumor-associated macrophages and enhancing infiltration by CD8+ T cells. Oncoimmunology.

[25] Pass HI, Lavilla C, Canino C, Goparaju C, Preiss J, Noreen S, Blandino G, Cioce M (2016). Inhibition of the colony-stimulating-factor-1 receptor affects the resistance of lung cancer cells to cisplatin. Oncotarget.

[26] Kogan M, Fischer-Smith T, Kaminsky R, Lehmicke G, Rappaport J (2012). CSF-1R up regulation is associated with response to pharmacotherapy targeting tyrosine kinase activity in AML cell lines. Anticancer Res.

[27] Yu W, Chen J, Xiong Y, Pixley FH, Dai XM, Yeung YG, Stanley ER (2008). CSF-1 receptor structure/function in MacCsf1r-/- macrophages: regulation of proliferation, differentiation, and morphology. J Leukoc Biol.

[28] Irvine KM, Burns CJ, Wilks AF, Su S, Hume DA, Sweet MJ (2006). A CSF-1 receptor kinase inhibitor targets effector functions and inhibits pro-inflammatory cytokine production from murine macrophage populations. FASEB J.

[29] Hu J, Nakano H, Sakurai H, Colburn NH (2004). Insufficient p65 phosphorylation at S536 specifically contributes to the lack of NF-kappaB activation and transformation in resistant JB6 cells. Carcinogenesis.

[30] Christian F, Smith EL, Carmody RJ (2016). The regulation of NF-κB subunits by phosphorylation. Cells.

[31] Bai D, Ueno L, Vogt PK (2009). Akt-mediated regulation of NFkappaB and the essentialness of NFkappaB for the oncogenicity of PI3K and Akt. Int J Cancer.

[32] Hohensinner PJ, Kaun C, Rychli K, Niessner A, Pfaffenberger S, Rega G, de Martin R, Maurer G, Ullrich R, Huber K, Wojta J (2007). Macrophage colony stimulating factor expression in human cardiac cells is upregulated by tumor necrosis factor-alpha via an NF-kappaB dependent mechanism. J Thromb Haemost.

[33] Wilson NJ, Cross M, Nguyen T, Hamilton JA (2005). cAMP inhibits CSF-1-stimulated tyrosine phosphorylation but augments CSF-1R-mediated macrophage differentiation and ERK activation. FEBS J.

[34] Stanley ER, Chitu V (2014). CSF-1 receptor signaling in myeloid cells. Cold Spring Harb Perspect Biol.

[35] Menke J, Kriegsmann J, Schimanski CC, Schwartz MM, Schwarting A, Kelley VR (2012). Autocrine CSF-1 and CSF-1 receptor coexpression promotes renal cell carcinoma growth. Cancer Res.

[36] Barbetti V, Morandi A, Tusa I, Digiacomo G, Riverso M, Marzi I, Cipolleschi MG, Bessi S, Giannini A, Di Leo A, Dello Sbarba P, Rovida E (2014). Chromatin-associated CSF-1R binds to the promoter of proliferation-related genes in breast cancer cells. Oncogene.

